# The long walk to African genomics

**DOI:** 10.1186/s13059-019-1740-1

**Published:** 2019-06-27

**Authors:** Serena Tucci, Joshua M. Akey

**Affiliations:** Lewis-Sigler Institute for Integrative Genomics, Carl Icahn Laboratory, Princeton University, South Drive, Princeton, NJ 08544 USA

African populations are underrepresented in genetics research, but two recent studies describing high-coverage whole-genome sequence data have provided new insights into African genetic diversity and population history.

Africa holds a special place in human evolution because it is the location where a number of hominin lineages, including modern humans, arose. Despite its crucial role in our evolutionary past, Africa remains largely underrepresented in genetic studies. Such underrepresentation limits our understanding of human evolution and population history, as well as the successful application of precision medicine to individuals of African ancestry. Two recent studies have helped to address some of these gaps by generating high-coverage genomes from 52 individuals, spanning 31 geographically and ethnically diverse African populations. The studies contribute to a more comprehensive and nuanced reconstruction of African population history and shed new light on modern human origins.

## The cradle of humanity

Fossil and genetic evidence indicates that modern humans evolved in Africa about 200 thousand years ago (kya), possibly as early as 300 kya [1]. Although several geographic regions have been proposed as potential ‘birthplaces’ of our species, there is now growing evidence to support the view that our species probably evolved within structured populations, connected to each other by gene flow across the whole African continent [2]. Thus, Africa has witnessed modern human evolution for much longer than anywhere else. The African continent is also the source of the massive demographic event, known as the ‘Out of Africa’ dispersal, which began around 60–100 kya and spread individuals, and their genes, outside Africa and throughout the world. Whether present-day people outside Africa descend from a single or multiple founding population(s), as well as the timing and routes of their dispersal, are still very much debated. Nonetheless, the signatures of our recent African origin persist in our genomes today. As modern humans dispersed from Africa, probably in small populations, they experienced a dramatic bottleneck. As a consequence, African populations today harbor more genetic diversity than any other population in the world, and the genetic diversity found in non-Africans represents only a subset of that found in Africa.

## Africa: a world of diversity

Africa is home to considerable cultural, linguistic, and genetic diversity. Indeed, more than 2000 ethno-linguistic groups are known to exist in Africa at present. The majority of languages spoken in Africa belong to four linguistic families: Niger-Kordofanian, Afroasiatic, Nilo-Saharian and Khoisan. The Khoisan linguistic family includes a highly diverse group of languages that share the use of click consonants, which are spoken by hunter-gatherer populations in southern Africa (referred to as the ‘San’) and by the Hadza and Sandawe in eastern Africa. Moreover, across the African continent, populations live in diverse environments, from tropical rainforests to deserts, and practice different subsistence strategies, such as agriculture, pastoralism, and some forms of hunting-gathering.

Despite the preeminent role that Africa played in human origins, past sequencing efforts have poorly captured the high level of diversity that exists in African populations. For example, the 1000 Genomes Project [3] has sequenced, at low coverage, the genomes of over 1000 currently living humans, including those of individuals from five indigenous African populations from Nigeria, Gambia, Sierra Leone and Kenya. While providing an incredible resource for examining genetic variation at a global scale, a much more comprehensive sampling of genetic variation in Africa is essential to provide an understanding of our past.

To this end, two new studies [4, 5] analyzed newly generated whole-genome sequences from 52 African individuals, spanning a broad range of ethnic, cultural, and linguistic groups, as well as populations practicing a range of subsistence strategies. Both studies found that geographic distance and linguistic affiliation play major roles in shaping patterns of population structure [6]. Hunter-gatherer populations in southern Africa, such as the Khoisan, and in the central African rainforest represent today the most diverse human populations worldwide. Among them, the Khoisan harbor the most divergent lineages and represent the basal group for all living humans. A recent study, based on ancient genomes derived from 2000-year-old remains from southern African hunter–gatherers, suggests that the divergence between the ancestors of present-day Khoisan groups and other populations might have occurred as early as 260–350 kya [7].

Both studies also captured signatures of some of the major dispersal events that shaped African population history. Indeed, the landscape of genetic variation in most sub-Saharan African populations appears to be dominated by traces of the so-called ‘Bantu expansion’. This migration of Bantu-speaking agriculturalists (linguistic family Niger-Kordofanian) originated around 4 kya in western-central Africa, and gradually spread throughout sub-Saharan Africa. In the course of their dispersal, Bantu-speaking groups admixed with the local hunter-gatherer populations that they encountered, to varying degrees, completely replacing them in some regions [8]. Bantu-related ancestry is also found in several present-day Khoisan and hunter-gatherer populations of the central African rainforests.

Genetic diversity in African populations was also impacted by the ‘back-to-Africa’ migration that brought genes from Eurasia back to the African continent. As a consequence, North African populations, represented in these two studies by the Mozabite, Saharawi, and Libyan people (who all speak Afroasiatic languages), show genetic affinities to Eurasian populations. Similarly, signatures of recent gene flow with Eurasian populations have also been found in eastern African populations. Further, it has been proposed that the subsequent southern spread of populations from eastern Africa might have brought East African- or Eurasian-related ancestry to southern Africa; the signatures of this demographic event persist in the genomes of modern-day Khoisan populations.

## Ghosts of human past

Modern humans overlapped for most of their history with other hominin groups, and an enduring point of contention is whether and the extent to which modern human ancestors admixed with other, now extinct, humans. DNA retrieved from Neandertal and Denisovan fossils revealed that our ancestors did admix with archaic hominin contemporaries, and remnants of Neandertal and Denisovan genomes can be found in contemporary individuals.

Although studies of archaic hominin admixture in Eurasian populations is progressing rapidly, comparable inferences in Africa are lacking. A challenge in studying hominin admixture in Africa is the lack of archaic reference genomes, which facilitate inferences of gene flow. To overcome this limitation, a recent study by Lachance and colleagues [9] applied a ‘fossil free’ statistical framework to detect putative archaic sequences without relying on an archaic genome. Applying this framework to 15 African genomes (from Hadza, Sandawe, and central African rainforest hunter–gatherers), the authors found evidence for admixture with an unknown hominin group. Following these observations, several studies have suggested that archaic hominin groups admixed with modern human ancestors in Africa, but the relationships of these archaic humans to the human lineage remain unsolved.

Lorente-Galdos et al. [5] used an Approximate Bayesian Computation (ABC) analysis to compare complex demographic models and to disentangle the source of putative introgression in the genomes of modern Africans. ABC is a flexible statistical framework that allows probabilistic model testing and parameter estimation for models where deriving the likelihood functions would be numerically intractable, and is a popular tool in population genetics. Some challenges remain, however, such as the issue of how to select the optimal set of summary statistics for a given model or parameter. To address this challenge, Lorente-Galdos et al. [5] generated informative summary statistics using a deep learning (DL) framework. Having identified an informative set of summary statistics, they used their approach to test six different demographic models, including admixture events with known and unknown hominin groups. Their results suggest that modern humans admixed with an unknown human population that diverged from the modern human lineage about 500 kya, The ‘vestige’ of this, now extinct, ghost population might still be lingering in the genomes of some sub-Saharan populations.

## Moving forward

Two new studies [4, 5] have analyzed newly generated datasets of African genomes, advancing our understanding of patterns of genetic diversity, population structure, and admixture in Africa. Yet more sampling efforts are needed to capture the high level of diversity that exists in African populations and to overcome the lack of ethnic diversity in human genomic research, which is currently dominated by studies of individuals of European ancestry [10]. Furthermore, the analysis of diverse populations is critical for delineating the genetic architecture of complex diseases, turning the promise of precision medicine into reality for all individuals, and for translating this knowledge into health care strategies.

Despite the poor conditions for DNA preservation, ancient DNA from Africa has also begun to inform African population history [7, 8]. The analysis of further ancient human remains from Africa will enable inferences of demographic events that occurred across the African continent, at different time scales. For example, ancient DNA would provide insights into patterns of genetic diversity in African populations that predate major demographic events, such as the Bantu expansion or the back-to-Africa migration.

Although modern and ancient genomes are becoming available, the development of new statistical frameworks that incorporate more complex scenarios will also be needed to make robust demographic inferences. For example, most coalescent-based methods commonly used to make inferences about population history, assume random mating. Recent studies show that deviations from this assumption generate spurious signals, which are often interpreted as changes in the sizes of populations [2]. Thus, ignoring population structure might result in inaccurate demographic inferences and caution is urged when interpreting this type of signal.

With the availability of high-quality genomes from Africa and the promise of many more, the opportunity now exists to add new chapters to the story of our African origins.

## Abbreviations

ABC analysis: Approximate Bayesian Computation (ABC) analysis
kya: Thousand years ago
